# Experience using donor human milk: A single‐center cohort study in Japan

**DOI:** 10.1111/ped.15071

**Published:** 2022-02-28

**Authors:** Kosuke Oikawa, Yuya Nakano, Tokuo Miyazawa, Yoshiyuki Hasebe, Haruhiro Kuwabara, Tomomasa Terada, Yumiko Sugishita, Akio Ebata, Mariko Takase, Hirotaka Ochiai, Akatsuki Kokaze, Katsumi Mizuno

**Affiliations:** ^1^ Department of Pediatrics Showa University School of Medicine Tokyo Japan; ^2^ Department of Hygiene, Public Health and Preventive Medicine Showa University School of Medicine Tokyo Japan

**Keywords:** donor human milk, enteral nutrition, infant, milk bank, very low birth weight

## Abstract

**Background:**

Donor human milk (DHM) became available in Japan when the first human milk bank was established in 2017. This study investigated the effects of DHM on enteral nutrition (EN) in very low birth weight (VLBW) infants in the single center in Japan.

**Methods:**

Seventy‐six VLBW infants hospitalized between April 2017 and March 2020 at Showa University Hospital were included in the study. We retrospectively evaluated age (hours) at which EN was initiated and age (days) until complete feeding (EN > 100 mL/kg/day) was achieved. We compared the DHM and non‐DHM groups, or the early human milk (EHM) and non‐EHM groups. The EHM group was defined as those in which EN was initiated with the mother’s own milk or DHM within 12 h of birth.

**Results:**

In 30 extremely low birth weight (ELBW) infants, EN was initiated at significantly earlier postnatal hours in the DHM group compared to those in the non‐DHM group. Complete feeding was achieved at significantly earlier ages in the EHM group after adjusting for gastrointestinal complications and gestational age. Additionally, the changes in body weight z‐scores from birth to term‐equivalent age were significantly greater in the EHM group after adjusting for exclusive breastfeeding and small for gestational age, compared to the non‐EHM group. Statistical significance was not noted in 46 subjects (birth weight, 1000–1500 g).

**Conclusion:**

The use of DHM may contribute to earlier initiation and achievement of EN, resulting in greater early postnatal growth in ELBW infants in Japan.

##  

There is a wealth of evidence supporting the health benefits associated with breastfeeding in premature infants^1^. However, there may be challenges in obtaining their mother’s own milk (MOM)^2^. Several international organizations and academic societies recommend the use of donor human milk (DHM) in premature infants when MOM is not available^3^, ^4^, ^5^; however, there were previously no accredited human milk banks in Japan. Therefore, the fasting period was prolonged or artificial milk was occasionally used instead of breast milk, which resulted in delayed establishment of enteral nutrition (EN)^6^.

The Japanese Human Milk Bank Association (JHMBA) was founded and Japan’s first human milk bank was established in Showa University Koto Toyosu Hospital in 2017, which facilitated the use of donor human milk (DHM) in premature infants. Feeding with DHM reduces the risk of complications such as necrotizing enterocolitis (NEC) in premature infants^7^, ^8^, ^9^. In addition, the time to full EN was significantly shorter in the DHM group than in the formula group in very low birth weight (VLBW) infants^10^, which prevented extrauterine growth restriction in premature infants^11^. However, In Japan, most neonatal intensive care units (NICUs) have not utilized DHM^12^. Consequently, Japan Pediatric Society recently recommended that DHM should be used as an alternative to MOM in preterm and VLBW infants^13^; however, there is limited information regarding its effects in Japan.

Since November 2018, we have frequently used DHM in VLBW infants admitted to the NICU at Showa University Hospital. The objective of this study is to investigate the effects of DHM on EN and early postnatal growth, as well as the incidence of complications due to the use of DHM in VLBW infants in the single center in Japan. This does not aim to compare using MOM and DHM, but to evaluate the benefits of using DHM instead of traditional methods (waiting for MOM or using formula when MOM is not available).

## Methods

### Subjects

The protocol of this study was approved by the Ethics Committee of the Showa University Graduate School of Medicine (reception number 3,173), and research information is available on the website https://www.showa‐u.ac.jp/albums/abm.php?d=505&f=abm00014771.pdf&n=3173OP.pdf. Moreover, the use of DHM at our facility was approved by the ethics committee (reception number 2,714). Written informed consent was obtained from the parents before using DHM.

Eighty‐eight VLBW infants admitted to the NICU at Showa University Hospital between April 2017 and March 2020 were included in this study. We retrospectively obtained information from medical records to evaluate the age (hours) at which EN was initiated, age (days) until complete feeding was achieved, changes in body weight *z*‐scores from birth to term‐equivalent age, and other perinatal factors such as gestational age, birthweight, and birthweight *z*‐scores. Infants whose weight and length at birth are less than the 10th percentile for gestational age were considered small for gestational age (SGA). This study defined initial EN as ≥ 0.5 mL per dose. Complete feeding was defined as achievement of EN to 100 mL/kg/day. The DHM group was defined as the group of infants who used DHM at least once after admission. Further, the early human milk (EHM) group was defined as the group in which EN could be initiated using MOM or DHM within the first 12 h of life. The non‐EHM group included infants in whom EN was not initiated within 12 h and those in whom EN was initiated with infant formula within 12 h. We defined term‐equivalent age as 40 weeks corrected gestation or discharge day from the NICU, if it was before 40 weeks corrected gestation.

We obtained information regarding whether gastrointestinal complications, such as Bell stage II NEC or higher, gastrointestinal perforation, and meconium‐related ileus, were observed during hospitalization. We also confirmed whether infants experienced other severe complications such as patent ductus arteriosus (requiring drug or surgical therapy excluding prophylactic indomethacin administration), late‐onset sepsis (with positive blood culture 72 h after birth), intraventricular hemorrhage (grade 3 or 4), retinopathy of prematurity (ROP) (requiring laser treatment and/or vitreous surgery), late‐onset circulatory collapse (clinical diagnosis), and chronic lung disease (requiring oxygen therapy at 36 weeks’ corrected gestation).

The exclusion criteria were: (A) <24 weeks gestation, (B) respiratory disorders requiring high oxygen levels (FiO_2_ > 60%), (C) circulatory insufficiency requiring a vasopressor, (D) chromosomal abnormalities, (E) major malformations or congenital cardiovascular malformation affecting hemodynamics, (F) hypoxic ischemic encephalopathy, (G) transfer to another hospital, (H) death, and (I) infants considered inappropriate for inclusion by the physician in charge.

### Our policy on nutritional management

Since November 2018, we routinely utilized DHM in VLBW infants when MOM was not available, except in infants whose parents refused it. Almost all VLBW infants born between November 2018 and March 2020 were fed MOM or DHM. We started to utilize DHM only in the early phase to initiate EN, but not in the later phase after EN was achieved. In contrast, VLBW infants born between April 2017 and October 2018 were fed MOM or infant formula for premature infants when MOM was not available.

This was a retrospective study, so there is no completely uniform nutrition protocol; however, we typically performed nutritional management as follows: (i) EN was initiated within 12 h of birth; (ii) intravenous nutrition was initiated with amino acids (2–3 g/kg/day) immediately after birth and then increased to 4 g/kg/day with EN in total and maintained unless the ammonia level increased; the fat emulsion was initiated at 0.5–1 g/kg/day (≥24 h after birth) and was gradually increased to a maximum of 2 g/kg/day; (iii) *Bifidobacterium breve* was administered daily; (iv) for breast milk fortification, HMS‐2^®^ (Morinaga Milk Industry Co., Ltd, Tokyo, Japan) was added initially with one‐quarter or one‐half of full fortification using 50–100 mL/kg/day of EN, followed by full fortification after a week. We considered discontinuing the use of fortifier or switching to HMS‐1^®^ (Morinaga Milk Industry Co., Ltd) at the start of oral feeding (~37 weeks corrected gestational age). The compositions of these two fortifiers are shown in Table S1. We occasionally considered administrating fentanyl or phenobarbital in the acute phase after birth for pain relief or sedation, often in extremely preterm infants born at less than 28 weeks' gestation.

### Statistical analysis

All analyses were performed with JMP^®^ version 15.0 (SAS Institute Inc. 100 SAS Campus Drive Cary, NC 27513‐2414, USA), and *P* < 0.05 was considered statistically significant. Subjects were classified into two subgroups based on birthweight: extremely low birthweight (ELBW) infants (birthweight, <1,000 g, ELBW group) and non‐ELBW infants (birthweight, 1,000–1,499 g, non‐ELBW group). Subgroups were assigned because each group had a different policy for EN initiation MOM or DHM was not available. Infant formula was used when MOM or DHM was not available in VLBW infants; however, it was not used in ELBW infants within 24 h after birth. We therefore performed statistical analyses for each group individually.

The Mann–Whitney *U*‐test was used to compare gestational age, birthweight, birthweight *z*‐scores, age (hours) at which EN was initiated, age (days) until complete feeding was achieved, and changes in bodyweight *z*‐scores from birth to term‐equivalent age between the DHM and non‐DHM groups or between the EHM and non‐EHM groups in both the ELBW and non‐ELBW groups. In addition, Fisher's exact test or Pearson's χ^2^ test was used to examine the differences in categorical data, such as the incidence of complications between each group. Multiple linear regression analysis was conducted to investigate the relationship between EHM and age (days) until complete feeding was achieved, after adjusting EHM feeding, gastrointestinal complications, and gestational age. It was also conducted to investigate the relationship between EHM and changes in bodyweight *z*‐scores from birth to term‐equivalent age, after adjusting EHM feeding, exclusive breast‐feeding (fed solely MOM and/or DHM) and small for gestational age.

## Results

Eighty‐eight VLBW infants (birthweight, <1,500 g) were born during the study period. Of these infants, 12 were excluded according to the exclusion criteria: A (two), D (three), E (four), F (one), G (one), and H (one). Consequently, 76 VLBW infants were evaluated in this study; their characteristics are shown in Table 1. Thirty ELBW infants and 46 non‐ELBW infants (birthweight, 1,000–1,499 g) were included. Seven infants had gastrointestinal complications due to meconium‐related ileus, and no infant had NEC. Among the 76 subjects in this study, 29 (38.1%) were SGA infants (15 in ELBW group and 14 in non‐ELBW group). Sixteen infants received fentanyl (15 in ELBW group and 1 in non‐ELBW group), and 16 infants (11 in ELBW group and 5 in non‐ELBW group) received phenobarbital for sedation during early postnatal life. No infants received morphine and midazolam, and no infants had a duodenal tube before the establishment of EN.

**Table 1:** Characteristics of subjects

	ELBW group (*n* = 30)	Non‐ELBW group (*n* = 46)	Total (*n* = 76)
Sex (male)	16	21	37
Gestational age (weeks)	26.7 (25.2, 29.7)	31.2 (28.9, 32.6)	29.7 (27.9, 32.1)
Birthweight (g)	747 (616, 886)	1,273 (1,157, 1,464)	1,120 (841, 1,311)
Birthweight z‐score	−1.60 (−2.73, −0.78)	−1.16 (−2.11, −0.19)	−1.31 (−2.35, −0.49)
Small for gestational age (*n*)	15	14	29
Use of fentanyl (*n*)	15	1	16
Use of phenobarbital (*n*)	11	5	16
The first EN (h)^†^	13.5 (9.0, 40.5)	10.5 (8.8, 17.3)	11.5 (9.0, 21.8)
Complete feeding (days)^‡^	13.0 (9.8, 19.5)	8.0 (7.0, 10.0)	9.5 (7.0, 12.8)
At the time of evaluation			
Corrected age (weeks)	40.0 (40.0, 40.0)	40.0 (40.0, 40.1)	40.0 (40.0, 40.1)
Bodyweight (g)	2,594 (1,987, 2,970)	2,779 (2,548, 3,155)	2,733 (2,445, 3,089)
Bodyweight *z*‐score	−1.59 (3.49, −0.39)	−0.97 (−1.63, 0.26)	−1.14 (−1.99, −0.02)
Changes in bodyweight z‐scores from birth to the time of evaluation	0.01 (−0.93, 0.82)	0.51 (0.15, 0.74)	0.45 (−0.24, 0.75)
Gastrointestinal complications (*n*)	6	1	7
Necrotizing enterocolitis (*n*)	0	0	0
Gastrointestinal perforation (*n*)	1	0	1
Meconium‐related ileus (*n*)/surgical cases (*n*)	6/3	1/0	7/3
Patent ductus arteriosus (*n*)/surgical cases (*n*)	17/10	6/0	23/10
Late onset sepsis (*n*)	3	0	3
Intraventricular hemorrhage (*n*)	1	0	1
Retinopathy of prematurity (*n*)	11	0	11
Late circulatory collapse (*n*)	4	0	4
Chronic lung disease (*n*)	16	2	18

Data are presented as numbers or medians (25th percentile, 75th percentile).

ELBW, extremely low birthweight; EN, enteral nutrition.

^†^ Age in hours when EN was initiated.

^‡^ Age in days until complete feeding was achieved.

The types of EN before or after the introduction of DHM are shown in Table S2. Of the 40 infants born before the introduction of DHM, 26 (65.0%), 13 (32.2%), and 1 (2.8%) were fed MOM, infant formula, and both MOM and formula, respectively, as the first EN. In contrast, of the 36 infants born after the introduction of DHM, 6 (16.7%), 27 (75.0%), 2 (5.6%), and 1 (2.8%) were fed MOM, DHM, both MOM and DHM, and both MOM and formula, respectively, as the first EN. Following the introduction of DHM, significantly more infants received DHM and significantly fewer infants received infant formula as the first EN (both *P* < 0.001). As a result, considerably more infants received EN within the first 12 h (*P* < 0.001). As shown in Table S3, the DHM group consisted of 34 VLBW infants, including 13 ELBW and 21 non‐ELBW infants, all of whom were born after the introduction of DHM in November 2018. On the other hand, the EHM group involved 28 infants, including 13 ELBW and 15 non‐ELBW infants. In the DHM and EHM groups, significantly more infants were born after the introduction of DHM in comparison with those born before the introduction of DHM (both *P* < 0.001). Before the introduction of DHM, only one infant (6.3%) in the ELBW group was fed infant formula as the first EN when MOM was not available, whereas 12 infants (50%) in the non‐ELBW group were fed infant formula. In contrast, after the introduction of DHM, no infants in both the ELBW and non‐ELBW groups were fed infant formula as the first EN when MOM was not available because we had the option of using DHM (data not shown).

### Extremely low birthweight group

The characteristics of ELBW infants in the DHM and non‐DHM groups, or EHM and non‐EHM groups, are shown in Table 2. There were no significant differences between the DHM and non‐DHM groups or the EHM and non‐EHM groups in sex, gestational age, birthweight, birthweight *z*‐scores, existence of sedation, and incidence of complications. The median age in hours at which EN was initiated was significantly earlier in the DHM group than in the non‐DHM group (9.0 and 30.0 postnatal hours in the DHM and the non‐DHM group, respectively; *P* = 0.001). Of the 13 infants who used DHM, 12 (92.3%) were able to start the first EN within 12 h of their birth.

**Table 2:** Comparison of parameters between the DHM and non‐DHM groups or between the EHM and non‐EHM groups in ELBW infants

	DHM group (*n* = 13)	Non‐DHM group (*n* = 17)	*P* ^§^	EHM group (*n* = 14)	Non‐EHM group (*n* = 16)	*P* ^¶^
Sex (male)	7	9	ns	7	9	ns
Gestational age (weeks)	27.3 (25.6, 29.0)	25.4 (24.9, 31.1)	ns	26.9 (25.4, 28.7)	26.4 (24.8, 31.1)	ns
Birthweight (g)	696 (612, 910)	750 (624, 880)	ns	674 (615, 907)	754 (623, 883)	ns
Birthweight z‐score	−1.88 (−2.56, −0.93)	−1.30 (−3.14, −0.72)	ns	−1.60 (−2.50, −1.00)	−1.70 (−3.27, −0.70)	ns
Small for gestational age (*n*)	6	9	ns	7	8	ns
Use of fentanyl (*n*)	7	8	ns	7	8	ns
Use of phenobarbital (*n*)	4	7	ns	4	7	ns
The first EN (h)^†^	9.0 (9.0, 10.5)	30.0 (16.0, 51.5)	0.001	9.0 (8.8, 10.0)	36.0 (21.3, 53.5)	<0.001
Complete feeding (days)^‡^	12.0 (9.0, 14.0)	18.0 (9.5, 23.0)	ns	11.5 (8.0, 14.0)	18.0 (10.3, 23.5)	0.043
Gastrointestinal complications (*n*)	2	4	ns	2	4	ns
Necrotizing enterocolitis (*n*)	0	0	ns	0	0	ns
Gastrointestinal perforation (*n*)	0	1	ns	0	1	ns
Meconium related ileus (*n*)/surgical cases (*n*)	2/1	4/2	ns/ns	2/1	4/2	ns/ns
Patent ductus arteriosus (*n*)/surgical cases (*n*)	6/3	11/7	ns/ns	6/3	11/7	ns/ns
Late onset sepsis (*n*)	1	2	ns	1	2	ns
Intraventricular hemorrhage (*n*)	0	1	ns	0	1	ns
Retinopathy of prematurity (*n*)	3	8	ns	4	7	ns
Late circulatory collapse (*n*)	2	2	ns	2	2	ns
Chronic lung disease (*n*)	6	10	ns	8	8	ns
At the time of evaluation						
Corrected age (weeks)	40.0 (40.0, 40.0)	40.0 (40.0, 40.1)	ns	40.0 (40.0, 40.0)	40.0 (40.0, 40.1)	ns
Bodyweight (g)	2,716 (2,521, 3,054)	2,405 (1,762, 2,955)	ns	2,730 (2,557, 3,067)	2,212 (1,717, 2,892)	0.028
Bodyweight *z*‐score	−1.14 (−1.81, −0.31)	−2.04 (−4.36, −0.60)	ns	−1.11 (−1.73, −0.21)	−2.66 (−4.47, −0.98)	0.025
Exclusive breast‐feeding (*n*)^††^	8	12	ns	10	10	ns
Changes in bodyweight *z*‐scores from birth to the time of evaluation	0.74 (−0.05, 1.37)	−0.61 (−1.11, 0.53)	0.009	0.78 (−0.02, 1.39)	−0.68 (−1.14, 0.34)	0.002

Data are presented as numbers or medians (25th percentile, 75th percentile).

DHM, donor human milk; EHM, early human milk; ELBW, extremely low birthweight; EN, enteral nutrition; ns, not significant.

^†^ Age in hours when EN was initiated.

^‡^ Age in days until complete feeding was achieved.

^§^ Comparison between the DHM and non‐DHM groups.

^¶^ Comparison between the EHM and non‐EHM groups.

^††^ Subjects fed solely mother's own milk and/or DHM.

On the other hand, the age in days until complete feeding was achieved was 11.5 and 18.0 days in the EHM and non‐EHM groups, respectively. Early human milk was significantly associated with earlier age in days until complete feeding was achieved (*P* = 0.043). This significance was strengthened after adjusting for gestational age and the presence of gastrointestinal complications in multiple regression analyses (*P* = 0.010), as shown in Table 3. In the model of age in days until complete feeding was achieved for an independent variable, it was significantly associated with gestational age and gastrointestinal complications (*P* < 0.001, respectively). The changes in bodyweight *z*‐scores from birth to term‐equivalent age were significantly greater in the EHM group than in the non‐EHM group (0.78 and −0.68 in the EHM group and the non‐EHM group; *P* = 0.002). The difference remained significant after adjusting for exclusive breast‐feeding and small for gestational age in multiple regression analyses (*P* = 0.002), as shown in Table 3.

**Table 3:** Factors associated with age (days) until complete feeding was achieved and changes in bodyweight z‐scores from birth to the time of evaluation in the ELBW group (*n* = 30)

Variables	Partial regression coefficient	Standardized partial regression coefficient	Standard error	*P* value
Age (days) until complete feeding was achieved				
EHM group	−3.128	−0.337	1.131	0.010
Gastrointestinal complications	6.342	0.547	1.406	<0.001
Gestational age	−1.502	−0.454	0.400	<0.001
Changes in bodyweight *z*‐scores from birth to the time of evaluation				
EHM group	0.794	0.546	0.232	0.002
Exclusive breast‐feeding^†^	−0.287	−0.187	0.246	0.253
Small for gestational age	0.251	0.173	0.231	0.287

The variables in this table were included as independent variables in the multiple linear regression models.

EHM, early human milk; ELBW, extremely low birthweight.

† Subjects fed solely mother’s own milk and/or donor human milk.

### Non‐ELBW group

Table S4 shows the characteristics of the infants in the DHM and non‐DHM groups. Only one infant in the non‐EHM group had a gastrointestinal complication (meconium‐related ileus). In contrast to that, in the ELBW group, the median postnatal ages (hours) at which EN was initiated in the DHM (10.0 h) and non‐DHM groups (11.0 h) were not significantly different. The median age (days) until complete feeding was achieved was not significantly different in the EHM group and the non‐EHM group (9.0 and 8.0 days, respectively). Similarly, the changes in bodyweight *z*‐scores from birth to term‐equivalent age were not significantly different in the EHM group and the non‐EHM group (0.46 and 0.51, respectively). The results remained unchanged after adjusting for confounding factors in multiple regression analyses (Table S5).

## Discussion

Many countries currently have active human milk banks.^14^, ^15^, ^16^, ^17^, ^18^ Since 2017, two human milk banks have been established in Tokyo, Japan; these banks transport DHM to hospitals in various areas in Japan as requested. However, there was previously no information regarding the effects of DHM on EN and early postnatal growth in VLBW infants, because most NICUs in Japan never utilized DHM. This study demonstrated that EN initiation was significantly earlier in the DHM group than in the non‐DHM group, and the earlier EN initiation was significantly associated with earlier achievement of complete EN and greater early postnatal growth from birth to term‐equivalent age in VLBW infants without increasing the incidence of complications. The findings of our study are crucial in that they may encourage the use of DHM as an alternative to MOM in VLBW infants in Japan, where there are different cultural and social attitudes toward DHM. Further, the incidence of NEC was comparatively low before the introduction of DHM in Japan.^19^

In this study, early EN was initiated within 12 h after birth in almost all ELBW infants (92.9%) after the introduction of DHM; however, it was initiated in only one ELBW infant (6.3%) before the introduction of DHM. Previous studies suggested that DHM or early EN initiation with DHM or MOM is associated with shorter duration of parental nutrition^20^ and earlier achievement of complete EN,^10^ which is consistent with our results. The effect of early trophic feeding on feed tolerance and short‐term growth has not been properly elucidated.^21^, ^22^ However, we postulate that the introduction of DHM is associated with earlier EN start, resulting in earlier achievement of complete EN and greater early postnatal growth because we typically waited for MOM to become available before using DHM in ELBW infants. In fact, these optimal effects of early EN initiation were not observed in non‐ELBW infants, who typically received EN with DHM or infant formula without waiting for MOM to become available, before and after the introduction of DHM. This protocol regarding EN initiation in ELBW infants appears identical to that used in other NICUs in Japan,^6^ so DHM may be useful for early EN initiation and achievement in ELBW infants.

A recent systematic review revealed that feeding with DHM resulted in a lower risk of developing NEC than feeding with formula.^23^ Previous studies have also suggested that DHM reduces the incidence of sepsis,^10^, ^24^ chronic lung disease (CLD),^24^, ^25^ and ROP;^10^ however, this has not been confirmed. In this study, there were no significant differences in the incidence of complications between the DHM and non‐DHM groups or the EHM and non‐EHM groups. No infant suffered from NEC, both before and after the introduction of DHM. We could not therefore evaluate the changes in the incidence of complications with low incidence because of the small number of subjects. Further investigation is required to evaluate the risk of these complications in a larger population.

### Limitations

This study had some limitations. First, we could not adjust for some confounding factors such as sex, birthweight *z*‐scores, sedation, and maternal factors due to the small number of subjects. The study subjects included several SGA infants. Some infants also received fentanyl and phenobarbital early postnatal life, which may influence feeding intolerance and incidence of meconium‐related ileus at early postnatal life. Second, information regarding the daily amount of EN for the study subjects was not available during this study; however, it may have influenced their postnatal growth. Third, this was a retrospective study conducted in a single institution. There might have been differences in the nutritional management of infants by the physicians in charge and treatment based on the time of birth (e.g., daytime, night‐time, holidays), although we had a general protocol for nutritional management, as previously mentioned. We also began using DHM for VLBW infants in our hospital in November 2018, and all subjects fed DHM were born after that. The comparison of DHM use and non‐use may be fairly close to that between cases before and after November 2018, although there were no particular changes in the management before and after.

### Conclusion

The use of DHM may contribute to earlier initiation and achievement of EN, resulting in improved early postnatal growth for ELBW infants. Further research is required to investigate the effects and safety of DHM in VLBW infants in a larger population.

## Disclosure

Katsumi Mizuno received consulting fees from the Japanese Human Milk Bank Association and support for attending meetings from Prolacta Bioscience Co., Ltd. The other authors declare no conflict of interest.

## Author contributions

K.O. and K.M. planned the study. Y.H., H.K., T.T., Y.S., A.E., and M.T. collected information from medical records. Y.N., H.O., and A.K. designed the study and examined its statistical validity. K.O., Y.N., T.M., and K.M. wrote the manuscript. All authors have read and approved the final manuscript.

## Supporting information


**Table S1**. Composition of Human Milk Fortifiers HMS‐1® and HMS‐2®.

**Table S2**. Type of enteral nutrition before or after introduction of DHM.

**Table S3**. Subgroup classification before and after introduction of DHM in the ELBW and non‐ELBW groups.

**Table S4**. Comparison of parameters between the DHM and non‐DHM groups or between the EHM and non‐EHM groups in non‐ELBW infants.

**Table S5**. Factors associated with age (days) until complete feeding was achieved and changes in body weight z‐scores from birth to the time of evaluation in the non‐ELBW group (*n* = 46).
