# Supplementary material for: Experience using donor human milk: A single‐center cohort study in Japan
Source: Pediatr Int. 2022 Feb 28;64(1):e15071. doi: 10.1111/ped.15071 (PMC9313846; doi:10.1111/ped.15071)
Supplement: Supplementary file 1 — Table S1. Composition of Human Milk Fortifiers HMS‐1® and HMS‐2®. [file PED-64-0-s003.pdf]

Supplementary Table 1. Composition of Human Milk Fortifiers HMS-1® and HMS-2®

| | HMS-1® | HMS-2® |
|---|---|---|
| Protein (g) | 0.7 | 1.0 |
| Fat (g) | 0 | 1.0 |
| Carbohydrate (g) | 1.5 | 1.8 |
| Energy (kcal) | 9 | 20 |
| Sodium (mg) | 9 | 18 |
| Potassium (mg) | 10 | 14 |
| Calcium (mg) | 70 | 100 |
| Phosphorus (mg) | 40 | 60 |

Contents added to 100 mL human milk.

HMS-1 and HMS-2® are bovine protein-derived breast milk fortifiers manufactured and sold by Morinaga Milk Industry Co., Ltd., Tokyo, Japan.
