# Supplementary material for: Experience using donor human milk: A single‐center cohort study in Japan
Source: Pediatr Int. 2022 Feb 28;64(1):e15071. doi: 10.1111/ped.15071 (PMC9313846; doi:10.1111/ped.15071)
Supplement: Supplementary file 2 — Table S2. Type of enteral nutrition before or after introduction of DHM. [file PED-64-0-s005.pdf]

Supplemental Table 2. Type of enteral nutrition before or after introduction of DHM

	Before introduction of DHM, n = 40 (%)	After introduction of DHM, n = 36 (%)	p-value
Type of the first EN [a]			
100% MOM	26 (65.0)	6 (16.7)	< 0.001
100% DHM	0 (0.0)	27 (75.0)	< 0.001
MOM and DHM	0 (0.0)	2 (5.6)	< 0.001
MOM and formula	1 (2.8)	1 (2.8)	ns
Formula	13 (32.2)	0 (0.0)	< 0.001
EN given in the first 12 h			
Yes	14 (35.0)	28 (77.8)	< 0.001
Type of EN			
MOM	4 (10.0)	6 (16.7)	ns
DHM	0 (0.0)	21 (58.3)	< 0.001
MOM and/or DHM	4 (10.0)	27 (75.0)	< 0.001
MOM and formula	1 (2.5)	1 (2.8)	ns
Formula	9 (22.5)	0 (0.0)	< 0.001

EN, enteral nutrition; MOM, mother's own milk; DHM, donor human milk; ns, not significant

[a] Not the first day of EN, but the first time of EN
