# Supplementary material for: Experience using donor human milk: A single‐center cohort study in Japan
Source: Pediatr Int. 2022 Feb 28;64(1):e15071. doi: 10.1111/ped.15071 (PMC9313846; doi:10.1111/ped.15071)
Supplement: Supplementary file 3 — Table S3. Subgroup classification before and after introduction of DHM in the ELBW and non‐ELBW groups. [file PED-64-0-s002.pdf]

Supplementary Table 3. Subgroup classification before and after introduction of DHM in the ELBW and non-ELBW groups

	Before introduction of DHM, n = 40 (%)	After introduction of DHM, n = 36 (%)	p-value
Total	40 (100)	36 (100)	ns
DHM group	0 (0.0)	34 (94.4)	< 0.001
EHM group	5 (12.5)	28 (77.8)	< 0.001
ELBW (< 1000 g of birth weight)	16 (40.0)	14 (38.9)	ns
DHM group	0 (0.0)	13 (36.1)	< 0.001
EHM group	1 (2.5)	13 (36.1)	< 0.001
Non-ELBW (1000-1499 g of birth weight)	24 (60.0)	22 (61.1)	ns
DHM group	0 (0.0)	21 (58.3)	< 0.001
EHM group	4 (10.0)	15 (41.7)	0.003

DHM, donor human milk; EHM, early human milk; ELBW, extremely low birth weight; ns, not significant
