# Supplementary material for: Experience using donor human milk: A single‐center cohort study in Japan
Source: Pediatr Int. 2022 Feb 28;64(1):e15071. doi: 10.1111/ped.15071 (PMC9313846; doi:10.1111/ped.15071)
Supplement: Supplementary file 4 — Table S4. Comparison of parameters between the DHM and non‐DHM groups or between the EHM and non‐EHM groups in non‐ELBW infants. [file PED-64-0-s001.pdf]

Supplementary Table 4. Comparison of parameters between the DHM and non-DHM groups or between the EHM and non-EHM groups in non-ELBW infants

	DHM group (n=21)	Non-DHM group (n=25)	p value [c]	EHM group (n=19)	Non-EHM group (n=27)	p value [d]
Sex (male)	11	10	ns	9	12	ns
Gestational age (weeks)	30.7 (28.4, 32.5)	31.6 (29.7, 32.9)	ns	30.9 (28.7, 32.6)	31.3 (29.3, 32.4)	ns
Birth weight (g)	1255 (1153, 1448)	1283 (1152, 1478)	ns	1255 (1163, 1467)	1306 (1142, 1463)	ns
Birth weight z-score	-0.98 (-1.61, 0.05)	-1.47 (-2.29, -0.53)	ns	-1.38 (-1.66, 0.25)	-1.10 (-2.35, -0.37)	ns
Small for gestational age (n)	5	9	ns	6	8	ns
Use of fentanyl (n)	1	0	ns	0	5	ns
Use of phenobarbital (n)	1	4	ns	0	1	ns
The first EN (hours) [a]	10.0 (9.0, 14.5)	11.0 (8.0, 24.5)	ns	9.0 (8.0, 10.0)	15.0 (9.0, 22.0)	< 0.001
Complete feeding (days) [b]	9.0 (7.0, 10.5)	8.0 (7.0, 9.5)	ns	9.0 (6.0, 11.0)	8.0 (7.0, 10.0)	ns
Gastrointestinal complications (n)	0	1	ns	0	1	ns
Necrotizing enterocolitis (n)	0	0	ns	0	0	ns
Gastrointestinal perforation (n)	0	0	ns	0	0	ns
Meconium related ileus (n) / surgical cases (n)	0 / 0	1 / 0	ns / ns	0 / 0	1 / 0	ns / ns
Patent ductus arteriosus (n) / surgical cases (n)	2 / 0	4 / 0	ns / ns	2 / 0	4 / 0	ns / ns
Late onset sepsis (n)	0	0	ns	0	0	ns
Intraventricular hemorrhage (n)	0	0	ns	0	0	ns
Retinopathy of prematurity (n)	0	0	ns	0	0	ns
Late circulatory collapse (n)	0	0	ns	0	0	ns
Chronic lung disease (n)	0	2	ns	1	1	ns
At the time of evaluation						
Corrected age (weeks)	40.0 (40.0, 40.1)	40.0 (40.0, 40.1)	ns	40.0 (40.0, 40.1)	40.0 (40.0, 40.1)	ns
Body weight (g)	2773 (2670, 3254)	2784 (2528, 3126)	ns	2764 (2674, 3057)	2827 (2511, 3205)	ns
Body weight z-score	-0.96 (-1.41, 0.54)	-0.98 (-1.71, 0.21)	ns	-1.11 (-1.46, -0.27)	-0.62 (-1.71, 0.33)	ns
Exclusive breastfeeding (n) [e]	9	5	ns	8	6	ns
Changes in body weight z-scores from birth to the time of evaluation	0.42 (-0.22, 0.64)	0.54 (0.29, 0.78)	ns	0.46 (-0.20, 0.66)	0.51 (0.28, 0.78)	ns

EN: enteral nutrition, DHM: donor human milk, EHM: early human milk, ELBW: extremely low birth weight, ns: not significant

[a] Age in hours when EN was initiated

[b] Age in days until complete feeding was achieved

[c] Comparison between the DHM and non-DHM group

[d] Comparison between the EHM and non-EHM group

[e] Subjects fed solely mother's own milk and/or DHM.

Data are presented as number or median (25th percentile, 75th percentile).
