# Supplementary material for: Experience using donor human milk: A single‐center cohort study in Japan
Source: Pediatr Int. 2022 Feb 28;64(1):e15071. doi: 10.1111/ped.15071 (PMC9313846; doi:10.1111/ped.15071)
Supplement: Supplementary file 5 — Table S5. Factors associated with age (days) until complete feeding was achieved and changes in body weight z‐scores from birth to the time of evaluation in the non‐ELBW group (n = 46). [file PED-64-0-s004.pdf]

Supplementary Table 5. Factors associated with age (days) until complete feeding was achieved and changes in body weight z-scores from birth to the time of evaluation in the non-ELBW group (n=46)

Age (days) until complete feeding was achieved	Partial regression coefficient	Standardized partial regression coefficient	Standard error	p value
Variables				
EHM group	0.312	0.117	0.337	0.360
Gastrointestinal complications	1.372	0.152	1.131	0.232
Gestational age	-0.695	-0.550	0.158	< 0.001
**Changes in body weight z-scores from birth to the time of evaluation**	**Partial regression coefficient**	**Standardized partial regression coefficient**	**Standard error**	**p value**
Variables				
EHM group	-0.210	-0.292	0.111	0.065
Exclusive breastfeeding [a]	0.020	0.026	0.125	0.874
Small for gestational age	0.093	0.123	0.122	0.448

EHM, early human milk; ELBW, extremely low birth weight

[a] Subjects fed solely mother's own milk and/or donor human milk.

The variables in this table were included as independent variables in the multiple linear regression models.
